# Role of galectin 3 binding protein in cancer progression: a potential novel therapeutic target

**DOI:** 10.1186/s12967-021-03085-w

**Published:** 2021-09-26

**Authors:** Emily Capone, Stefano Iacobelli, Gianluca Sala

**Affiliations:** 1 Department of Innovative Technologies in Medicine and Dentistry, University of Chieti-Pescara, 66100 Chieti, Italy; 2 Center for Advanced Studies and Technology (CAST), Via Polacchi 11, 66100 Chieti, Italy; 3 Mediapharma Srl, Via Colonnetta 50/A, 66100 Chieti, Italy

## Abstract

The lectin galactoside-binding soluble 3 binding protein (LGALS3BP) is a secreted, hyperglycosylated protein expressed by the majority of human cells. It was first identified as cancer and metastasis associated protein, while its role in innate immune response upon viral infection remains still to be clarified. Since its discovery dated in early 90 s, a large body of literature has been accumulating highlighting both a prognostic and functional role for LGALS3BP in cancer. Moreover, data from our group and other have strongly suggested that this protein is enriched in cancer-associated extracellular vesicles and may be considered a promising candidate for a targeted therapy in LGALS3BP positive cancers. Here, we extensively reviewed the literature relative to LGALS3BP role in cancer and its potential value as a therapeutic target.

## Introduction

LGALS3BP (a.k.a. Gal-3BP, 90 K, Mac2-BP) is a secreted multifunction glycoprotein present in human body fluids. Glycoproteins are a class of proteins containing glycans linked to amino acid side chains, and they play an important role in cancer because cellular transformation is typically accompanied by changes in protein glycosylation. The major types of changes in protein glycosylation associated with neoplastic transformation include changes in O-glycans (GalNAc-Ser/Thr) and N-glycan [1, 2]. Changes in protein glycosylation can result in altered glycoprotein conformation, oligomerization, and turnover and can also be associated with altered cell signaling pathways, like proliferative signaling, resistance to cell death, evasion of growth suppression, genome instability and mutation, angiogenesis, invasion and metastasis, tumor-promoting inflammation, and immune evasion [1, 2]. Functionally, LGALS3BP role has been mainly investigated in two contexts: neoplastic transformation and innate immunity. Although most of the investigated functions are related to the secreted form of the protein, LGALS3BP possesses intracellular activity, mainly implicated in the regulation of the innate immune responses. Indeed, it was demonstrated that intracellular LGALS3BP reduced the amount of HIV Gag at the plasma membrane via interaction with vimentin and inhibits the proteolytic maturation of HIV gp160/Env [3]. Furthermore, intracellular LGALS3BP has a role in the prevention and treatment of inflammatory diseases by suppressing TAK1-dependent NF-κB activation [4] and regulates centriole biogenesis and centrosome hypertrophy in cancer cells [5].

LGALS3BP was first discovered from two independent research groups as a 90KDa tumor-associated antigen recognized by SP2 monoclonal antibody in CG-5 human breast cancer cells and by L3 monoclonal antibody in Calu-1 human lung cancer cells [6–8]. In parallel, it was identified as a novel ligand of beta-galactoside binding lectin galectin-3 (formerly Mac-2) [9, 10]. On the other hand, LGALS3BP expression was found to be induced by viral infection and in response to a variety of cytokines unleashed by inflammatory processes, including IFN-α (Interferon), IFN-β, IFN-γ, TNF-α (Tumor Necrosis Factor). In the recent past, a prominent role for LGALS3BP in tumor progression and spreading has been elucidated, and a growing body of evidence is accumulating supporting the notion that this multifunction hyperglycosylated protein is a drive force in different processes leading to cell transformation. In this context, data collected so far indubitably document that high LGALS3BP expression levels in tissues and serum are associated with unfavorable clinical outcomes in a wide variety of malignancies, including breast [6, 11–25] and lung [8, 26–29] cancer, melanoma [10, 30–36], ovarian [37–42], hepatocellular [36, 43–46], pancreatic [47–50], prostatic [51, 52] and oral squamous cell [53–59] carcinomas, neuroblastoma [60–63], glioblastoma [64, 65], gastric cancer [66] and lymphoma [67]. However, it was proposed that LGALS3BP is associated with a favorable clinical outcome in colorectal carcinoma [68–80], pleural mesothelioma [81] and Ewing’s sarcoma [82].

This review summarizes the current knowledge of structural features of LGALS3BP, its pattern of expression in cancer and the potential role as novel therapeutic target in cancer.

### Structure

Human LGALS3BP has been purified from culture medium of tumor cell lines, serum and milk and shown to be a large non-covalent oligomeric protein with a molecular mass of ~ 1000 kDa [7, 8, 83]. The sequence of human LGALS3BP was elucidated after cDNA cloning [83, 84] and found to code for a 567-amino acid sequence preceded by a leader peptide of 18-amino acids at the N-terminus. This signal peptide is important in guiding the protein into the secretory pathway and it is then involved in a proteolytic cleavage during the maturation process of the protein. In fact it is absent in the mature secreted form of LGALS3BP [7–9, 85]. The protein has seven potential *N*-linked glycosylation sites and three potential O-glycosylation sites and is composed of four domains. Following the signal peptide sequence, it contains a scavenger receptor cysteine-rich (SRCR) domain (Domain 1), making it as a member of the SRCR protein superfamily [3]. This domain is an ancient and highly conserved domain of about 110 residues and does not induce aggregation [3]. In addition to the SRCR domain, LGALS3BP harbors a BTB/POZ (Broad-Complex, Tramtrack and Bric a brac/Poxvirus and Zinc finger) domain and a BACK (BTB and C-terminal Kelch) domain, which constitute domain 2 and 3 and are important in mediating oligomer formation and interaction with extracellular matrix. BTP/POZ domains often self-associate as dimers, tetramers, or even large oligomers [3]. Indeed, purification and scanning electron microscopy of LGALS3BP showed high molecular mass (> 1000 kDa) oligomers, prominently dodecamers, with ring-shaped structures of 30–40 nm in diameter [85, 86]. Domain 4, consisting of a 26-kDa C-terminal sequence, is inactive; but it has been hypothesized that proteolytic processing of the C-terminal region of LGALS3BP may have regulatory functions [3, 83].

**Fig. 1:**
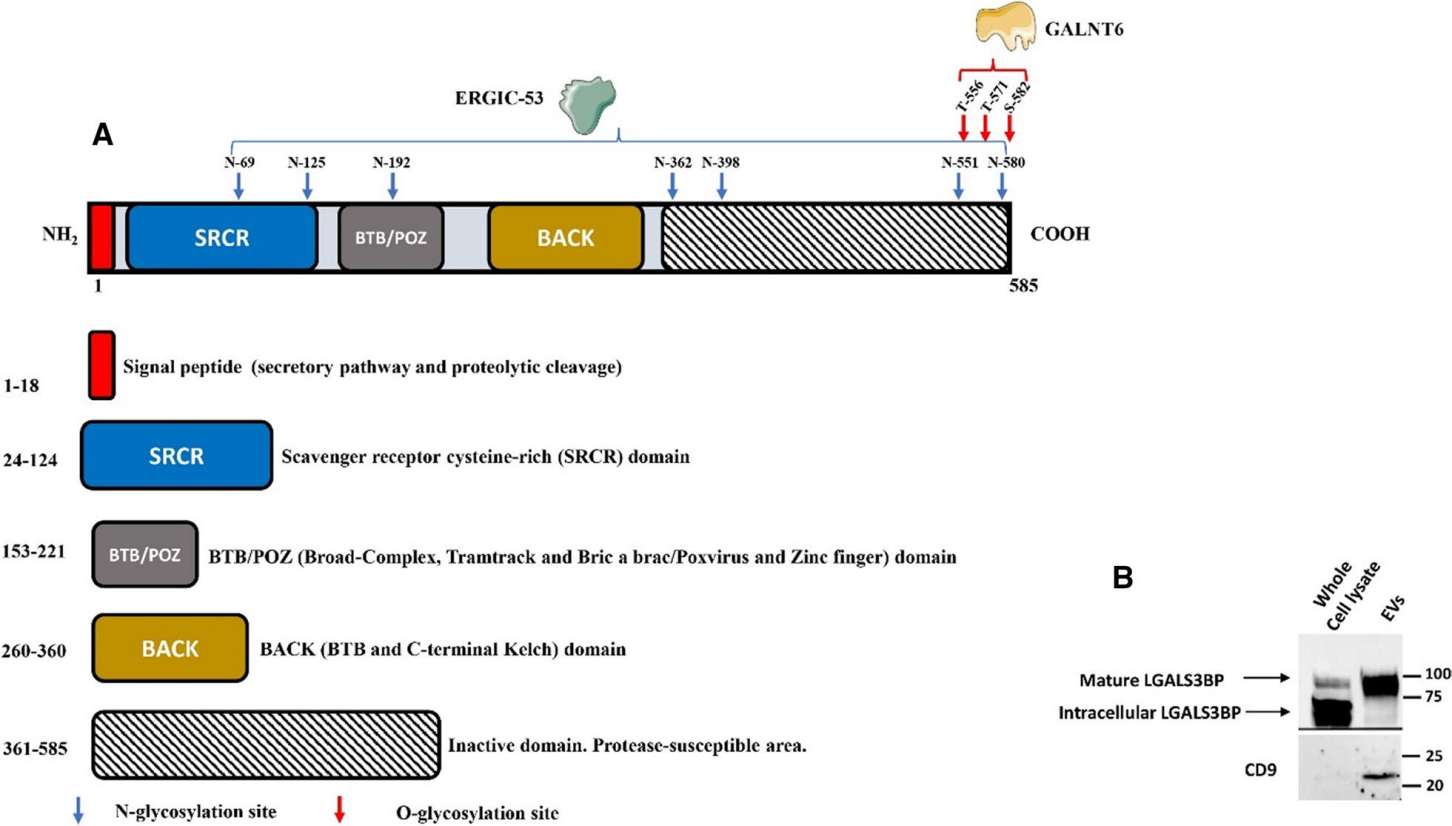
Structure and domains of LGALS3BP. **A** Schematic representation of LGALS3BP domains. N and O glycosylation sites and proteins involved in LGALS3BP modification are indicated. N: Asparagine; T: Threonine; S: Serine. **B** LGALS3BP intracellular and mature forms from neuroblastoma SKNAS whole cell lysate and isolated EVs are shown. CD9 was used as marker for purified EVs preparation

Purified LGALS3BP from cell lysates and culture media as well as human milk and serum, has three distinct forms sizing 90, 75 to 25 kDa; in fact, it can be proteolytically cleaved by plasmin and another endogenous protease in two forms of 75 and 25 kDa, representing fragments of N- and C-terminal sequence, respectively. Anyway, the secreted form of the protein usually appears as a 90–100 kDa protein in SDS-PAGE (Sodium Dodecyl Sulfate-Polyacrilamide Gel Electrophoresis), and treatment with N-glycosidases reduces the molecular mass to the calculated value of LGALS3BP monomer (65.3 kDa), indicating the presence of extensive glycosylation in all seven N-glycosylation sites. Terminal sialic acid and fucose are present in some forms of LGALS3BP [3, 8, 10, 42, 60, 83, 85, 87]. The secretion of LGALS3BP involves an interaction with ERGIC-53 (ER Golgi intermediate compartment 53-kD protein), an endoplasmic reticulum-Golgi transporter molecule that recognizes high-mannose type N-glycans, this means that N-glycosylation of LGALS3BP appears to be essential for its secretion [88]. Binding studies in solid-phase assays demonstrated β1 integrin-mediated cell adhesion for LGALS3BP, as well as interactions with collagens IV, V and VI, fibronectin and nidogen which were consistent with an extracellular matrix localization [85]. Structure, domains, mature and intracellular forms of free and EVs-associated LGALS3BP are represented in Fig. 1.

### Pattern of expression and role of LGALS3BP in human cancer

#### Breast cancer

Breast cancer patients sera and breast cancer cell culture media were among the first sources for identification and purification of LGALS3BP [6, 11]. In a preliminary study, it was observed that patients with metastatic breast cancer display elevated LGALS3BP serum levels in 51.3% of cases, and that high LGALS3BP levels were significantly associated with the occurrence of metastases to liver, a shorter DFS (Disease Free Survival) and a younger age [12]. The prognostic role of LGALS3BP was also evaluated by immunohistochemistry in tumor tissues of a consecutive series of node-negative breast cancer patients. The study showed a strong association between high LGALS3BP expression and decreased DFS, DRFS (Distant Recurrence-Free Survival) and OS (Overall Survival) in two independent series of node-negative breast cancer patients who didn't receive systemic therapy after surgery [13]. These data were further confirmed in a report showing a significative association between LGALS3BP and occurrence of metastasis in a cohort of 249 ER (Estrogen Receptor)-negative breast cancer patients [14].

Other studies attempted to understanding the molecular mechanism underlying the negative prognostic role of LGALS3BP in breast cancer. A first report showed that adhesion of ZR-75-1 breast cancer cells to endothelial cells was mediated by endothelial E-selectin and LGALS3BP, thus identifying this latter protein as a novel E-selectin ligand. This suggested a primary role of LGALS3BP in cell adhesion and cancer metastasis, explaining the poor prognosis of breast cancer patients with LGALS3BP overexpressing tumors. It appeared that expression of LGALS3BP by cancer cells was critically regulated, and an optimal expression level was required for evading the immune response and to colonize distant tissues [15]. Another intriguing study on prometastatic mechanism was proposed by White et al. [16]. To metastasize, tumor cells often need to migrate through a layer of collagen-containing scar tissue which encapsulates the tumor, constituted by monocyte-derived fibrocytes, a collagen-secreting profibrotic cells. They found that LGALS3BP secreted by the human metastatic breast cancer cells MDA-MB-231 inhibited monocyte-derived fibrocyte differentiation, and, conversely, galectin-3 promoted monocyte-derived fibrocyte differentiation. Therefore, LGALS3BP and galectin-3 were proposed as new modulators of fibrosis in the tumor microenvironment. In line with this, increased levels of tumor cell associated LGALS3BP were observed at the leading edge of breast cancer biopsies. To make consistent LGALS3BP as an indicator for poor prognosis and metastasis in breast cancer (and other epithelial cancers), the role of the protein on *adherens junctions* and invasive cell motility was evaluated. It was demonstrated that LGALS3BP destabilizes E-cadherin–p120-catenin complex through ubiquitination proteasomal degradation, promoting the release and motility of cancer cells from tumor tissues through the weakening of cell–cell *adherens junctions* [17]. This finding provided a mechanistic explanation of distant recurrence in cancer patients exhibiting high serum LGALS3BP levels. Additionally, beyond its prometastatic role, LGALS3BP secreted by breast cancer cells may function critically as a pro-angiogenic factor through a dual mechanism: the induction of VEGF (Vascular Endothelial Growth actor) expression in human breast cancer cells by activation of the PI3K/Akt pathway, and stimulation of endothelial cell tubulogenesis in a VEGF-independent, galectin-3- dependent manner [18]. Thus, extracellular LGALS3BP may dock galectin-3 molecules, and the resulting complex cross-links and clusters the integrin on the surface of the endothelial cells, thus promoting activation of FAK-mediated signaling pathways, which in turn modulate the angiogenic cascade [18]. Finally, the role of LGALS3BP O‑glycosylation was evaluated in terms of breast cancer growth. In fact, when the protein was O‑glycosylated at T556, T571 and S582 positions by GALNT6 (N-acetylgalactosaminytransferase 6), it was able to promote autocrine cell growth. On the contrary, when Ala substitutions occurred in these thee glycosylation sites, GALNT6‑dependent LGALS3BP O‑glycosylation and secretion were drastically reduced, resulting in suppression of autocrine growth‑promoting effect [19].

Reports also demonstrated that LGALS3BP is an IFN-inducible gene. Both IFN-α, IFN-β and IFN-γ enhanced mRNA and protein secretion in breast cancer cells [20, 21]. Moreover, administration of rIFN-α-2b to breast cancer patients significantly increased LGALS3BP serum levels over pre-administration values [22]. Finally, Noma et al. [23] investigated the role of NF-kB (Nuclear Factor kappa-light-chain-enhancer of activated B cells) in the adhesion of breast cancer cells to a substrate and revealed that adhesion was suppressed through inhibition of NF-kB-regulated LGALS3BP expression. Cloning of LGALS3BP promoter confirmed the presence of regulatory binding sites for IFN and NF-κB [24, 25].

### Lung cancer

Calu-1 human lung cancer cells were among the first cell lines where LGALS3BP was identified as an antigen recognized by L3 antibody [8].

A study investigated the role of LGALS3BP expression, evaluated by IHC (Immunohistochemistry) as an adverse prognostic indicator in 72 pathological stage I non-small cell lung cancer patients [26]. High expression levels were observed in 20 of the 72 (28%) tumors and the results were confirmed by quantifying the protein by ELISA (Enzyme-Linked Immunosorbent Assay). Moreover, patients whose tumors expressed high LGALS3BP, displayed a significant lower DFS and OS rates compared to patients with low LGALS3BP expression tumors. Incidence of distant metastases was also higher in patients with high LGALS3BP expressing tumors [26]. Another study reported that 87.5% of lung carcinoma cell lines and 60.7% of tumor tissues expressed high levels of LGALS3BP mRNA and that there was a correlation of LGALS3BP protein with clinical stage (stage I and II: 27.8%; stage III and IV: 60%) [27].

Using a novel antibody library-based proteomic technology to identify lung cancer-associated secreted functional biomarkers, Sun et al. identified LGALS3BP as a potential therapeutic target and biomarker for lung cancer [28]. They analyzed LGALS3BP serum levels from 320 lung cancer patients and 80 healthy donors, confirming the potential value of this protein as a serological marker for lung cancer. In fact, the diagnostic power of LGALS3BP seemed to be superior to four tumor markers for lung cancer, including CEA (Carcinoembryonic Antigen), Cyfra21–1 (Cytokeratin fragment), and NSE (Neuron-Specific Enolase). In addition, the concentrations of LGALS3BP was an independent prognostic factor significantly correlating with tumor histology (it was higher in sera derived from small cell lung cancer patients compared to non-small cell lung cancer patients), lymph node and distant metastases [28].

Finally, LGALS3BP appeared to be involved in resistance to 17-AAG (17-N-Allylamino-17- demethoxygeldanamycin), an HSP90 inhibitor tested in phase II/III clinical trials in lung cancer [29]. Indeed, data from this report suggested that LGALS3BP mediates the resistance to 17-AAG through PI3K/Akt activation, both in vitro and in vivo [29].

### Melanoma

Together with breast and lung cancer, melanoma was one of the first malignancies where LGALS3BP was originally detected [30, 31]. Initially, the protein was identified as a cytoplasmic melanoma-associated antigen (CYT-MAA) and considered to be different from LGALS3BP. Years later, the evidence they were interchangeably recognized by 465.12S and SP2 monoclonal antibodies, lead to the conclusion that CYT-MAA and LGALS3BP were indeed a unique protein [32, 33]. Regarding clinical significance, a first study examined LGALS3BP serum level in 128 melanoma patients during immunotherapy. Before start of therapy, 63% of patients showed elevated serum LGALS3BP levels, in particular patients with stages IIb to IV disease. After initiation of the therapy, the levels of LGALS3BP declined in more than 90% of patients and below the positive cut-off of normal range in 56% of cases within 5 months. By contrast, there was no decline in LGALS3BP serum level in 11 melanoma patients who served as untreated controls. In conclusion, measurement of LGALS3BP serum level appeared to show potential as an early marker of prognosis in patients with stages IIb to IV melanoma and, mostly, an intermediate marker of response in these patients [32]. A complementary study from the same research group measured the serum levels of LGALS3BP in 117 melanoma patients stratified into four risk group based on the stage of the disease, and it was confirmed that LGALS3BP may serve as a prognostic marker for clinical outcome in melanoma patients treated with immunotherapy [34].

From the biological viewpoint, A375 melanoma cells served as a model to demonstrate LGALS3BP mediated cell–cell adhesion and homotypic cell aggregation via bridging galectin-3 on the cell surface in a specific carbohydrate-dependent manner [9]. Also, this cellular model was used to demonstrate galectin-1 induced multicell aggregation in a carbohydrate-dependent manner which is mediated, at least in part, by LGALS3BP [35]. Finally, evidence was provided showing that LGALS3BP interacting with integrins α_5_β_1_, α_v_β_1_ and α_6_β_1_ mediates cell adhesion in the melanoma cell line C8161 [36]. On the basis of these findings, cellular effects of LGALS3BP- mediated integrin adhesion can be divided as ‘early and late’: early (1–3 h) effects comprised survival and proliferation signaling transduction via Akt, JNK and the Ras cascade; while late effects appeared after sustained (44 h) cellular exposure to LGALS3BP and resulted into increased cellular motility, migration and viability. Importantly, LGALS3BP was demonstrated to bind one or more integrins depending on their availability within a particular cell type, and this unique integrin expression profile could also explain why LGALS3BP is capable of providing adhesion to some but not all cancer cell types [36].

### Ovarian cancer

There are several studies demonstrating the prognostic relevance of LGALS3BP in ovarian cancer. First, serum levels of LGALS3BP and CA125 (Cancer Antigen 125) were found to be elevated in 73 ovarian cancer patients and 70 patients with benign gynecological conditions, respectively, and the sensitivity increased to 86% when a combination of the two markers was used. LGALS3BP expression rate positively correlates with tumor differentiation grade and with recurrence disease during chemotherapy. These data suggested that LGALS3BP combined with CA125 may be used for the detection and monitoring of ovarian cancer [37]. In another study, it was found that LGALS3BP and s-IL-2R (soluble form- Interleukin 2 Receptor) serum levels were associated with poor prognosis in a retrospective study on 152 ovarian-cancer patients before primary surgery [38]. Many years later, a research group performed tandem-mass spectrometry analysis of *secretome* derived from early-stage 3D models of ovarian cancer in order to discover novel biomarkers. Subsequently, the top five candidate biomarkers, including LGALS3BP were validated by immunohistochemistry on TMAs (Tissue MicroArrays) and survival analyses in a large series of > 200 primary early stage ovarian cancer tissues. LGALS3BP was found expressed in 43% of stage I/II tumours and 62% of stage III/ IV tumours and positively associated with tumour recurrence [39].

Moreover, connection between LGALS3BP and IFN was confirmed also in ovarian cancer. Evidence was provided that both IFN-α and IFN-γ upregulate the level of mRNA expression and the secretion of LGALS3BP in 3 ovarian carcinoma cell lines [40]. Another report showed that release of LGALS3BP was significantly increased by treatment with IFN-γ in ovarian cancer cells, while neither IL-1β (Interleukin-1β) nor TNF-α treatment consistently influenced the secretion of LGALS3BP [41]. It’s finally to be noted that the presence of LGALS3BP int the compartment of cancer-derived extracellular vesicles (EV)s was for the first time identified in ovarian cancer [42]. A proper chapter on this topic will follow in the review.

### Hepatocellular carcinoma

A research group investigated prognostic significance of LGALS3BP in hepatocellular carcinoma (HCC) in several studies. First, high LGALS3BP serum levels in HCV (Hepatitis C Virus) -infected cirrhotic patients (one of the major cause of HCC) and in HCC patients compared to the control group were observed [43, 44]; then, they retrospectively investigated the prognostic significance of serum LGALS3BP values in 40 HCC cases at first diagnosis, and on the basis of these results they assumed that serum LGALS3BP and AFP (alpha-fetoprotein) levels could be very useful not only for the diagnosis of HCC but also for its prognostic evaluation. In fact, HCC patients with serum LGALS3BP below the cut-off of normal range showed an improved overall survival and the mean LGALS3BP levels were significantly higher in HCC patients with more than one lesion [45]. To clarify the biological role of the LGALS3BP, the same group evaluated the ability of two monoclonal antibodies SP-2 and 1A4.22, to reveal this glycoprotein in both serum and tissue from HCC patients. Tissue expression of LGALS3BP was detected by IHC in 20 HCC patients, while the LGALS3BP serum level was assessed by the ELISA assay in 13 HCC patients. Positive staining was seen only in the epithelial cells and was cytoplasmic and granular in all instances [46]. At molecular level, data were provided showing that, along with melanoma, also in the hepatocellular carcinoma Hu-H7 adhesion on LGALS3BP is mediated by integrins α_1_β_1_ and α_v_β_1_ [36].

### Pancreatic cancer

First evidence about the potential role of LGALS3BP in pancreatic cancer derived from a study by Künzli et al. in which authors analyzed, both at mRNA and protein level the expression and cellular distribution in human pancreatic carcinoma tissues of three ligands for galectin-3, Lamp-1, Lamp-2 (Lysosomal-associated membrane proteins 1–2), and LGALS3BP [47]. Results from this work revealed either increased Lamp-1, Lamp-2, and LGALS3BP gene transcription or increased mRNA stability in pancreatic carcinomas in vivo, and at protein level, primary carcinoma samples and their corresponding lymph node metastasis overexpressed all three ligands concomitantly in the pancreatic carcinoma cells. However, there was no correlation between LGALS3BP mRNA expression levels in the tumor tissue and prognosis [47]. Subsequent studies focused on N-glycosylation profile of LGALS3BP in pancreatic cancer using proteomic approaches. One of these [48], revealed that the core protein expression of LGALS3BP was elevated 2.5-fold in pancreatic tumor tissue compared to normal pancreas, and its N-glycopeptides were also significantly up-regulated in PDAC (Pancreatic Ductal Adenocarcinoma), with over tenfold and threefold increase compared to normal pancreas and chronic pancreatitis. In parallel, there was overexpression of their partners galectin-1 and galectin-3. So they hypothesized that the implication of LGALS3BP N-glycosylation in pancreatic tumorigenesis was possibly through intensifying the specific interplay between LGALS3BP and galectins to mediate cell−cell and cell-extracellular matrix interaction, angiogenesis, and apoptosis of tumor cells [48]. The second report [49], using a spectral library-based proteomic approach, analyzed N-glycosylated peptides of various protein including LGALS3BP in a cohort consisting of patients with PDAC, chronic pancreatitis and healthy subjects. They revealed that the plasma level of *N*-glycosylated LGALS3BP peptides -representing specific glycosylation sites- were frequently elevated in the early stage PDAC. Each of the *N*-glycosylated peptide plasma level (AAIPSALDT**N**SSK, ALGFE**N**ATQALGR and DAGVVCT**N**ETR) was found to be elevated in 59% of the cases in the PDAC group compared to controls [49]. Finally, in a recent manuscript Samonig et al. [50] compared pancreatic tumor initiating cells (TICs) isolated from three-dimensional tumor spheroid cultures with non tumor-initiating cells (non-TICs) enriched in planar cultures employing differential proteomics. LGALS3BP, S100A8 and S100A9 (S100 calcium-binding protein A8 and A9) (two known antimicrobial peptides) have been identified as the most differentially expressed proteins, suggesting a potential role for these proteins regarding cancer stemness in pancreatic TICs.

### Prostatic cancer

Role of LGALS3BP was investigated in several studies. In a work published in 2006 by Bair et al. [51], LGALS3BP prognostic value was evaluated trough expression pattern analysis in 300 prostate patient tissue samples. Results obtained from this study showed that LGALS3BP was mostly lost in PIN (prostatic intraepithelial neoplasia), while it was overexpressed in 38.4% of analyzed prostate cancer tumor samples. Moreover, using LNCaP cell model researcher suggested that LGALS3BP induced IL-6 release which, in turn upregulated promatrilysin (pro-MMP-7), a matrix metalloproteinase responsible of degradation of many ECM (Extracellular Matrix) proteins and whose expression is crucial in prostate cancer [51]. Years later, comparative proteomic technologies were used to separate by two-dimensional electrophoresis (2-DE) total proteins from 10 cases of prostate cancer, benign prostatic hyperplasia and normal prostate tissue [52]. The screening revealed that a total of 18 proteins were differentially expressed and identified by mass spectrometry and database searches. Interestingly, among these markers, LGALS3BP resulted to be the most significant differential expressed in prostate cancer samples. According with this findings, functional analysis demonstrated that LGALS3BP overexpression is correlated with the occurrence, proliferation, differentiation and metastasis of cancer cells [52].

### Oral squamous cell carcinoma (OSCC)

The first study analyzing LGALS3BP expression in oral squamous cell carcinoma (OSCC) was conducted from Weng et al. [53] in 2008. These authors were searching potential biomarkers in OSCC using 1D SDS-PAGE combined with MALDI-TOF Mass Spectrometry. They analyzed the secretome of two OSCC cell lines (OEC-M1 and SCC4) and LGALS3BP was identified for the first time in the group of OSCC-related proteins. It was overexpressed in 76% of 146 OSCC tissue specimens analyzed by IHC and its levels in blood samples of OSCC patients, measured by ELISA, resulted to be higher compared to healthy controls [53]. Finally, to investigate the possible role of LGALS3BP in OSCC cells, they used RNA interference-based knock-down and revealed for the first time that LGALS3BP was involved in regulating growth and motility of OSCC cells [53].

Role of LGALS3BP in cancer progression of OSCC was indirectly confirmed by Endo et al. [54]. In this study, they performed methylation-based screening and genome-wide gene expression profiling in combination with a prediction database analysis in 18 OSCC cell lines, and identified a novel tumor-suppressive microRNA *miR-596* directly targeting LGALS3BP*.* Therefore, they revealed that both overexpression of *miR-596* and knockdown of LGALS3BP suppressed cell proliferation and induced apoptosis in OSCC cell lines, suggesting that LGALS3BP might act as an oncogene in this type of tumour [54]. A new mechanism involving LGALS3BP was proposed in 2018 by Fukamachi et al. [55], who demonstrated that, in OSCC patients, overexpression of MCFD2 (Multiple Coagulation Factor Deficiency protein) correlated with higher risk of regional lymph node metastasis occurrence, and that this overexpression seemed to occur via secretion of LGALS3BP.

More recently, a study showed that LGALS3BP was overexpressed in a cohort of 92 OSCC tissues, and this overexpression was associated with unfavorable clinical characteristics in terms of OS, RFS (Recurrence Free Survival) and DFS [56]. They also proposed that LGALS3BP promoted OSCC cell proliferation and migration by activation the PI3K/AKT axis [56]. LGALS3BP was also found in elevated concentration in the saliva of OSCC patients by two research groups [57, 58]. The first group aimed to validate IL-1β, IL-8 and LGALS3BP as salivary biomarkers for OSCC, enrolling 117 Indian patients, grouped into subcategories of 31 early (TNM stage I-II) and 27 late-stage OSCC (TNM stage III-IV), 30 PMOD (Potentially Malignant Oral Disorders) and 29 post-treatment patients. LGALS3BP showed to be discriminatory between early and late stage OSCC as well as early stage OSCC + PMODs and controls [57].The other research study, instead, found LGALS3BP in Taiwan high-risk population using as proteomic approach liquid chromatography multiple reaction monitoring mass spectrometry (LC/MRM-MS) [58]. Oropharyngeal squamous cell carcinoma (OPSCC) is another common type of cancer developing in the Head and Neck region, which starts from the oropharynx (the throat area at the back of the mouth). Human papilloma virus (HPV), and in particular the high-risk genotype HPV16, is an established etiological factor for oropharyngeal squamous cell carcinoma (OPSCC), with nearly 90% of HPV-positive OPSCCs. Dickinson et al. [59] used a label-free quantitative mass spectrometry methodology which could discriminate between the HPV + OPSCCs, and those that are HPV-independent. Of 174 serum proteins identified, complement component C7 (C7), apolipoprotein F (ApoF) and LGALS3BP significantly increased in HPV-positive tumors; this result was not unexpected as its expression is upregulated in both chronic and acute viral infections, as reviewed by Loimaranta et al. [3].

### Neuroblastoma

Researchers from De Clarke’s lab published two studies on LGALS3BP role in neuroblastoma a few years apart from each other. In the first study, they identified and isolated LGALS3BP from serum-free conditioned medium of several neuroblastoma cells, and proved that it stimulated expression of IL-6 followed by an increase in Erk1/2 activation in human bone marrow stromal cells (BMSC) expressing its receptor Galectin-3 [60]. IL-6 in turn induced proliferation of neuroblastoma cells, creating a favorable microenvironment for progression of metastatic neuroblastoma. Years later, the same group went deeper into the mechanism and identified a distal region of IL-6 promoter that contained 3 CCATT/enhancer binding protein (C/EBP) binding domains involved in the transcriptional upregulation of IL-6 by LGALS3BP [61]. They also confirmed that Galectin-3 present in BMSC interacted with LGALS3BP, playing an important role in LGALS3BP/Gal-3/ Ras/MEK/ERK signaling pathway. Finally, they demonstrated that tumor-stromal cells axis plays a role not only in the bone marrow microenvironment but also in primary tumors where tumor-associated macrophages are the source of IL-6 among others and this was consistent with the observation of an abundant presence of LGALS3BP in primary human tumor specimens in proximity of IL-6 expressing cells [61].

In parallel, confirmation of prognostic role of LGALS3BP in neuroblastoma came from the work by Morandi et al. [62], in which expression and secretion of LGALS3BP was evaluated in neuroblastoma cell lines, primary tumors and metastatic neuroblasts, and protein serum levels compared in neuroblastoma patients versus age-matched healthy children. They showed that LGALS3BP was expressed and secreted by neuroblastoma cell lines and, more importantly, by metastatic neuroblasts isolated from bone marrow samples. Importantly, they found higher protein concentration in sera from neuroblastoma patients compared to healthy children, which significantly correlated with a higher incidence of relapse. In conclusion, these results validated LGALS3BP as marker of treatment response and as potential target for neuroblastoma immunotherapy, given its immunogenicity [62]. Additionally, neuroblastoma was the cancer context in which LGALS3BP was identified as extracellular ligand of endosialin (also known as Tumor Endothelial Marker 1, TEM-1) [63]. This study also revealed that the expression patterns of endosialin and LGALS3BP were mutually exclusive respectively in the tumor stroma and in the tumor cells; and that this interaction invoked repulsion in controlling tumor cell–stromal cell crosstalk, strongly suggesting important roles during tumor progression and metastasis.

### Glioblastoma multiforme (GBM)

In 2014, a research group [64] used the bio-orthogonal chemical reporter strategy (BOCR) in combination with label-free quantitative mass spectrometry (LFQ-MS) in order to identify cell surface sialoglycoproteins differentially expressed in several human primary GBM cell cultures compared with fetal and adult astrocytes and neural progenitor cells (NPCs) isolated from human brain. Among the differential expressed 801 glycoproteins, they found LGALS3BP for the first time reported associated to glioblastoma [64]. Several years later, another study confirmed the upregulation of Galectin-3 and LGALS3BP at both RNA and protein level in GBM tissues and were associated with shorter overall survival in GBM patients, in particular in the pro-neural subtype [65].

### Gastric cancer

There is only a study that investigated LGALS3BP role in gastric cancer by Park et al. [66]. In this work, authors demonstrated that LGALS3BP expression was upregulated by human telomerase reverse transcriptase (hTERT), whose activity is peculiar in gastric cancer. Moreover, they showed that LGALS3BP was overexpressed in gastric tumors both as secreted protein and at immunohistochemical level, and it was significantly associated with distant metastases and later tumor stages, appearing to be a good prognostic marker for gastric cancer [66].

### Lymphoma

Role of LGALS3BP in lymphoma was evaluated in one work from Fornarini et al. [67], where serum level of the protein was measured by ELISA in 137 patients. Results from this study showed that high levels of LGALS3BP is associated with a lower rate of treatment response compared to low LGALS3BP levels. Moreover, they proved a role for LGALS3BP in mediating lymphoma cell resistance to chemotherapy. Indeed they showed that high production and deposition of LGALS3BP by lymphoma cells in extracellular matrix protected tumor cells against cytotoxic drugs [67].

### Colon carcinoma

Role and significance of LGALS3BP in colon carcinoma is more controversial, as some studies have been published showing data in support of the hypothesis that in this contest the protein has tumor promoting properties and others demonstrating antitumoral mechanisms of LGALS3BP. One of the first studies focused on the analysis of galectin-3 expression in human adenomas and adenocarcinomas, found that its ligand, LGALS3BP, was increased in the blood plasma of patients with both adenomatous and adenocarcinomatous lesions [68]. Another research group identified a 100 kDa glycoprotein secreted by colon carcinoma cell lines as LGALS3BP modified with poly-N-acetyllactosamine structures [69, 70]. In a second work, due to the importance of cell-type-specific glycosylation, they have chosen to focus on isolated protein from the conditioned medium of HT-29 colon carcinoma cells infected with recombinant vaccinia virus expressing LGALS3BP-Hys as model for their functional studies [71]. LGALS3BP was demonstrated to interact with various extracellular matrix proteins including laminin-1, laminin-5, laminin-10, fibronectin, collagen IV, as well as galectin-3; and although to a lesser extent with collagen I and galectin-1; while, in contrast to previous findings, it was unable to mediate adhesion of seven colon cancer cell lines and a sampling of normal cell lines, suggesting that LGALS3BP produced by HT- 29 cells is a poor cell-adhesive substratum [71]. Going deep in understanding LGALS3BP involvement in galectin-3- mediated cell adhesion, authors were able to demonstrate for the first time its dual concentration-dependent role. In fact, at high concentration, inhibited galectin-3- mediated HT-29 cell binding but at lower concentrations, it enhanced cell binding. Hence, they speculated that the relative ratio between LGALS3BP and galectin-3 secreted by colon cancer cells in vivo may play an important role during colon cancer progression and metastasis by modulating tumor cell adhesion [71]. Starting from these assumptions, scientific community was divided into two research lines. In particular, Iacovazzi et al. [72] and Wu et al. [73] found that LGALS3BP concentrations in colorectal cancer patients at an advanced stage were significantly higher than those at an early stage, clarifying a positive correlation of colon cancer with LGALS3BP. In 2011, a report discovered the dendritic cell (DC)-specific intercellular adhesion molecule- 3-grabbing non-integrin (DC-SIGN) as a novel interactor of LGALS3BP in colorectal cancer (CRC). Indeed, using affinity chromatography and mass spectrometry authors identified DC-SIGN, which is a type II transmembrane C-type lectin expressed on myeloid dendritic cells and monocyte-derived dendritic cells (MoDCs) often associated to CRC tissues [74]. Importantly, they identified specific glycan structures (α1-3,4-fucose moieties) on LGALS3BP needed for its interaction with DC-SIGN, which resulted to be cancer specific, as they were not found in the protein isolated from healthy donors sera [74].

Finally, in support of the same thesis and proposing a novel mechanism by which LGALS3BP might influence immune cell activation, a study published in 2014 by Läubli et al. [75] revealed that, in LS180 colorectal cancer cells, LGALS3BP was a ligand of several types of CD33-related subset acid-binding immunoglobulin-like lectins (Siglecs), often associated with the modulation of immune response to cancer. In particular, they found that LGALS3BP interacted with Siglec-9, but also recognized Siglec-5 and Siglec-10, thus orchestrating the inhibition of different immune cells, including NK (Natural Killer) cells and neutrophils [75].

A different line of research proposed LGALS3BP as a tumor suppressor in CRC. A first study suggested a functional interaction between LGALS3BP and KITENIN (KAI1 C-terminal interacting tetraspanin, a metastasis-promoting gene). Authors proposed that the antitumor effects of KITENIN knockdown observed in CRC cancer cells might be derived from the generation of tumor-specific immune response through increment of LGALS3BP secretion from non-immune tumour cells [76]. In a second report from the same group, scientists found that LGALS3BP inhibits Wnt pathway by promoting the degradation of β-catenin via ISGylation-dependent proteasomal-ubiquitination. Moreover, authors revealed that this antitumor activity was mediated by the interaction of LGALS3BP with CD9/CD82 complex at the CRC cells membrane [77]. Finally, in a successive work, the same research group aimed to identify the β-catenin domain responsible of this mechanism. Results from this study demonstrated that LGALS3BP promotes degradation of mutant β-catenins lacking the ISGylation or phosphorylation sites [78]. Role of LGALS3BP in inducing β-catenin degradation was also confirmed in a study from Pikkarainen et al. [79]. Here, authors unveiled that LGALS3BP interacts with CD9/CD82 tetraspanins leading to ubiquitin-dependent degradation of β-catenin and suppression of Wnt signaling, thus explaining another mechanism through which LGALS3BP may exert its antitumor activity in CRC.

Finally, in 2015 Piccolo et al. [80] confirmed and extended these findings, demonstrating that LGALS3BP expression in primary tumor tissue correlated with a better disease-free and overall survival outcome, whereas low LGALS3BP expression correlated with a poorer survival outcome; moreover they showed that LGALS3BP silenced human CRC cells by RNAi formed large tumors when implanted in nude mice and that intra-tumor delivery of human recombinant LGALS3BP induced regression of established CRC xenografts [80].

### Mesothelioma

Human pleural malignant mesothelioma (MM) is a particular type of cancer that involves the serosal lining of the pleural cavity. A research group showed that the mean LGALS3BP level was significantly higher in MM pleural effusions than the levels detected in lung cancer or benign pleural disease patients. The difference in LGALS3BP expression between MM and lung cancer was confirmed by immunostaining, but without a significative difference between early and advanced stage disease. On the other hand, high serum LGALS3BP levels were shown to correlate with increased survival in MM patients. Results suggested that LGALS3BP expression at serum and pleural effusions level does not have the same biological significance, in contrast with previous studies in other tumors; maybe because some tumour-specific factors could affect systemic and local expression of LGALS3BP [81]. Overall, knowledge is limited only to this study, and further studies should clarify LGALS3BP role in mesothelioma.

### Ewing’s sarcoma

Another type of cancer in which LGALS3BP seemed to have a favorable prognostic value is Ewing’s sarcoma (EWS). A study from Zambelli et al. showed that LGALS3BP, together with IFITM2 (Interferon-Induced Transmembrane Protein 1) and STOML2 (Stomatin-Like Protein 2) expression is associated with a favorable outcome and a lower risk of developing metastasis [82]. This association between LGALS3BP and prognosis was confirmed both at mRNA and protein level in tumor tissues but not in serum level, suggesting a role for LGALS3BP in proximity of local tumor microenvironment. Engineered enhancement of LGALS3BP expression in EWS cells resulted in inhibition of anchorage independent cell growth and reduction of cell migration and metastasis, while silencing of LGALS3BP expression reverted these pro-tumoral cell behaviors, thus providing further functional validation of expression data obtained in clinical samples [82].

**Fig. 2:**
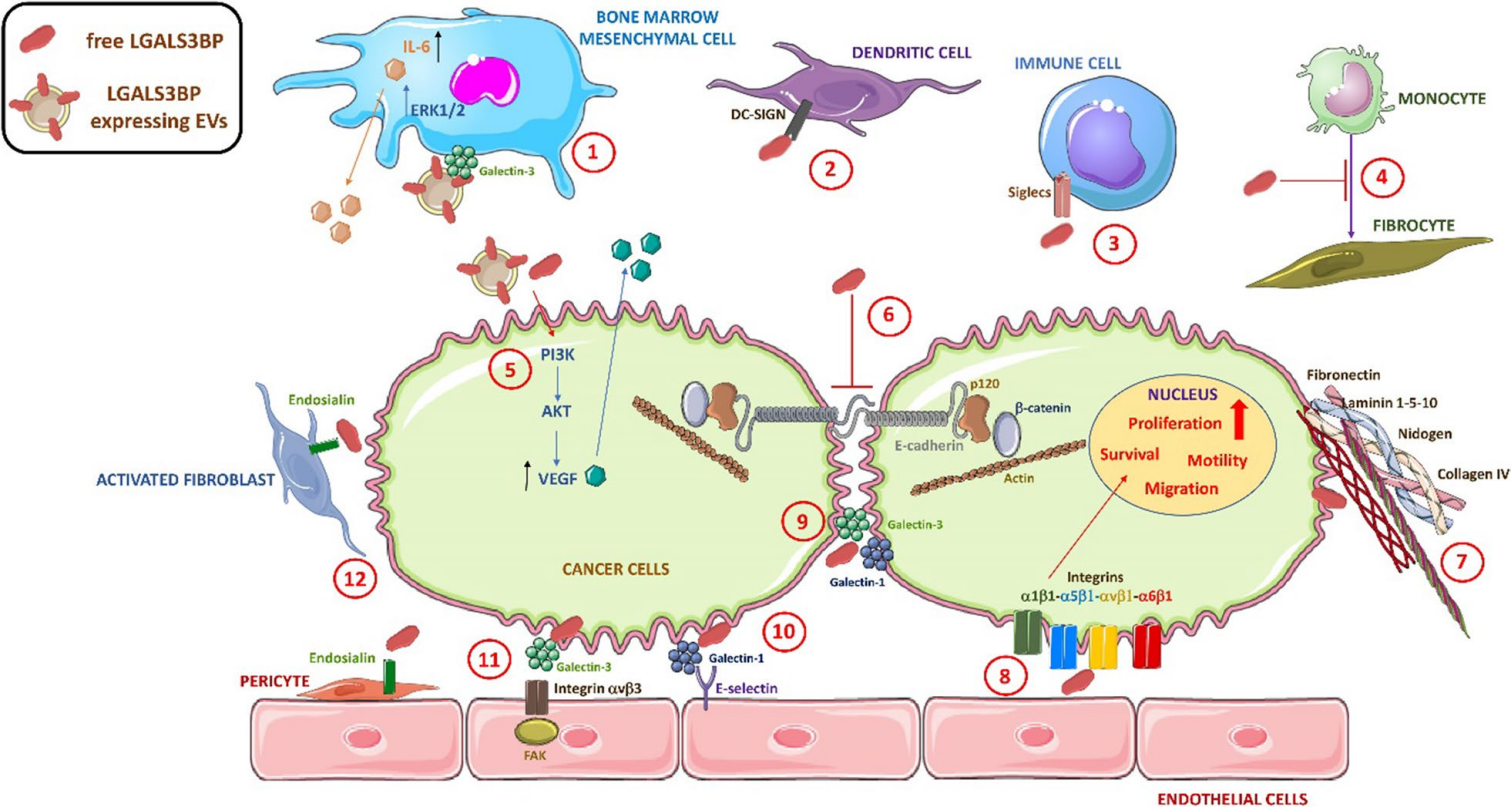
Schematic representation of proposed LGALS3BP functions in cancer. **1.** EVs-associated LGALS3BP induces IL-6 production from bone marrow mesenchymal cells contributing to the creation of pro-tumorigenic microenvironment [60, 61, 94]. **2.** LGALS3BP interacts with DC-SIGN to suppress Dendritic Cells maturation [74]. **3**. LGALS3BP interacts with Siglecs receptor on immune cells stimulating immune-evasion [75]. **4.** LGALS3BP is involved in inhibition of monocyte-fibrocyte differentiation [16]. **5.** Free and EVs-associated LGALS3BP induces VEGF production in cancer cells via PI3K/AKT signaling promoting angiogenesis [18, 97]. **6**. LGALS3BP destabilizes *adherens junctions* of cancer cells via dissociation of the E-cadherin–p120-catenin complex [17]. **7.** LGALS3BP mediates adhesion of cancer cells to several matrix extracellular proteins like fibronectin, nidogen, laminin 1-5-10 and collagen IV [71, 85]. **8**. LGALS3BP has a role in the integrins-mediated adhesion of cancer cells to endothelial cells, promoting survival, proliferation, motility and migration pathways [18, 36, 100]. **9.** LGALS3BP mediates homotypic aggregation cell induced by galectin-1 and galectin-3 [9, 35]. **10.** LGALS3BP is involved in E-selectin mediated adhesion to endothelial cells [14, 15]. **11.** LGALS3BP promotes angiogenesis through binding to endothelial integrins and FAK activation mediated by galectin-3 [18]. **12.** LGALS3BP has a role in cross-talk between tumor and stroma mediated by endosialin expressed on pericytes and activated fibroblasts [63]

Patient pattern expression, prognostic values and LGALS3BP functions are summarized in Table 1 and Fig. 2. In Table 1, for each type of cancer, worldwide incidence and mortality available rates are reported [89].

**Table 1:** LGALS3BP expression and prognostic significance in human cancers

Cancer incidence and mortality rate (%)	Samples and analysis	Prognostic significance	References
Breast cancer (11.7%–6.9%)	I. IHC analysis in 2 independent set of primary tumors derived from patients with resected unilateral cancer without nodal involvement: Training set (N = 170)—Validation set (N = 120) II. IHC analysis in 249 invasive breast cancer patients	I. LGALS3BP high expression predicts worse DFS (p < 0.007), DRFS (p < 0.008) and OS (p < 0.01) II. LGALS3BP high expression predicts earlier development of distant metastasis and shorter patient survival in the ER-negative group (p = 0.0353) and not in ER-positive group	[13] [14]
	I. Sandwich-type ELISA of serum samples from 185 patients with breast cancer, 56 patients with benign breast disease and 165 healthy donors II. Sandwich-type ELISA of serum samples from 425 breast cancer patients with no evidence of disease after surgery (NED), 310 patients with metastasis and 285 healthy female blood donors	I. Abnormal LGALS3BP levels are present in a greater percentage of breast cancer patients compared to healthy controls and benign breast disease patients. High LGALS3BP correlates with tumor staging (p < 0.01), and with tumor burden, as its levels decreases after surgical removal II. LGALS3BP expression level is higher in breast cancer patients compared to healthy donors, and it positively correlates with shorter OS in node-positive NED patients (p = 0.004). Regarding patients with developed metastatic disease, there is a positive correlation with metastatic liver involvement (p = 0.009), a shorter disease-free interval (p = 0.005) and a younger age (p = 0.01)	[11] [12]
Lung cancer (11.4%–18%)	I. IHC analysis of surgically resected stage I NSCLC (Non-Small Cells Lung Cancer) tumors from 72 patients II. IHC analysis of primary lung carcinoma tissues resected from 28 at the time of surgical removal	I. LGALS3BP high expression is strongly associated with chance of developing distant metastasis at various time intervals from complete resection. LGALS3BP positively correlates with shorter 5-year DFS and OS (p = 0.0001 and p = 0.0003, respectively) II. Protein expression of LGALS3BP is strongly associated with clinical staging of lung carcinoma, although difference is not statistically significant	[26] [27]
	I. ELISA analysis of serum samples from 320 lung cancer patients and 80 healthy donors	I. Serum concentrations of LGALS3BP in lung cancer patients are significantly higher than those in healthy controls (p < 0.05). Positive correlation with tumor histology (p = 0.000), lymph node metastases (p = 0.022), and distant metastases (p = 0.001). LGALS3BP concentrations above cut off value (6 mg/ml) correlate with poor OS (p = 0.000)	[28]
Melanoma (1.7%–0.6%)	I. Sandwich-type ELISA of serum samples from 128 patients with melanoma, of which 117 treated with immunotherapy and 11 with placebo II. Sandwich-type ELISA on sera collected from 117 randomly selected malignant melanoma patients, who were enrolled into melanoma therapy trials	I. LGALS3BP high expression predicts risk of recurrence or progression (p = 0.0079), and represents a potential early marker for response to immunotherapy II. Elevated LGALS3BP expression is associated with tumor stage and age and predicts increased risk of melanoma progression during follow-up (p = 0.03)	[32] [34]
Ovarian cancer (1.6%, 2.1%)	I. IHC analysis of ~ 210 primary early-stage (stage I/ II) ovarian cancer tissues of mixed histologies organized in two TMAs	I. LGALS3BP expression is heavily associated with stage tumor (p < 0.0001), higher risk of tumour recurrence (p = 000001) and with optimal debulking status (p = 0.018) as well as patient age (p = 0.005). I Positive correlation with worse OS is found but not with statistical significance (p = 0.075)	[39]
	I. Sandwich-type ELISA on sera collected from 73 ovarian cancer patients and 70 patients with benign gynaecological conditions II. Sandwich-type ELISA on serum samples from 152 patients, who underwent primary surgery for epithelial ovarian cancer	I. Compared to patients with benign ovarian tumors, LGALS3BP serum level is significantly higher in ovarian cancer patients. Statistically significant correlation of high LGALS3BP expression with grade of tumor differentiation and chemotherapy response II. Serum antigen LGALS3BP levels are positively correlated with residual disease and tumor grade (p < 0.05). LGALS3BP concentrations above cut off value (6.3 U/ml) correlate with an unfavourable clinical course (p < 0.03)	[37] [38]
Hepatocellular carcinoma (4.7%–8.3%)	I. Sandwich-type ELISA on serum samples from 11 chronic active hepatitis (CAH), 48 liver cirrhosis and 36 HCC patients II. Sandwich-type ELISA on serum samples from 172 patients, of which 103 patients with liver cirrhosis and 69 patients with HCC III. Sandwich-type ELISA on serum samples from 40 cirrhotic HCC at first diagnosis	I. According to a cut-off point of 14 mg/mL for LGALS3BP, increasing positivity is observed from CAH to liver cirrhosis and HCC II. Abnormally high serum levels of LGALS3BP are present in a greater percentage of HCC patients compared to cirrhosis patients, often associated with anti-HCV antibodies presence III. LGALS3BP levels are significantly higher in HCC patients with more than one lesion. Patients with serum LGALS3BP below the cut off value (14 ng/ml) show improved OS (p = 0.02)	[44] [43] [45]
Pancreatic cancer (2.6%, 4.7%)	I. IHC analysis of pancreatic carcinoma tissues from 28 patients undergoing a partial duodenopancreatectomy (Whipple resection)	I. LGALS3BP is strongly expressed, but no significant prognostic correlation is observed	[47]
Prostatic cancer (7.3%, 3.8%)	I. IHC analysis of TMA containing four morphologically representative areas of tissue from 300 patients, of which 286 had evidence of prostate cancer	I. LGALS3BP is over-expressed in prostate cancer tissues and mostly lost in PIN, but no significant clinical correlation is observed	[51]
Oral squamous cell carcinoma (2.0%, 1.8%)	I. IHC analysis of tissues derived from 146 OSCC patients II. IHC analysis of tissues derived from 92 OSCC patients	I. LGALS3BP expression significantly correlates with the differentiation status of OSCC (p = 0.006), with the highest positive rate seen in cases of well-differentiated OSCC II. Elevated expression levels of LGALS3BP are significantly associated with poorer clinicopathological features, including higher T stages (p = 0.043), more recurrence (p = 0.002) and shorter OS (p < 0.0001)	[53] [56]
	I. Sandwich-type ELISA on serum samples collected from 91 OSCC patients and 106 healthy controls II. ELISA on saliva samples collected from 117 various stages OSCC subjects (including 30 cases of PMODs and 42 control subjects matched according to age, sex and socioeconomic status	I. Serum levels of LGALS3BP are significantly higher in OSCC patients versus healthy controls (p < 0.0001), but this is not statistically associated with various clinicopathological characteristics, although patients with well-differentiated carcinoma or advanced clinical stage tend to have higher LGALS3BP levels II. LGALS3BP is a highly significant indicator in OSCC stage I-II patients and high risk PMODs with p = 0.0008 and p = 0.0001, respectively; but is not discriminatory for late stage OSCC	[53] [57]
Neuroblastoma	I. Sandwich-type ELISA on serum samples collected at diagnosis from 47 patients with different stages of neuroblastoma disease and 38 controls samples collected from age-matched healthy children, admitted for accidental traumatic injuries	I. LGALS3BP concentration is significantly higher in sera from neuroblastoma patients than in sera from healthy children (p = 0.0005). LGALS3BP serum levels above the cut off level (91.47 ng/ml) significantly correlate with a higher incidence of relapse (p = 0.032)	[62]
Glioblastoma multiforme (1.6%, 2.5%)	I. IHC analysis of 508 primary GBM tumor cases and 10 adjacent normal tissue cases	I. LGALS3BP is significantly upregulated in GBM tissues compared with adjacent normal tissues and its high expression significantly correlates with shorter OS (p = 0.013)	[65]
Gastric cancer (5.6%, 7.7%)	I. IHC analysis of 22 samples of gastric cancer tissues	I. LGALS3BP expression is higher in cancerous tissues compared with the normal mucosal tissues. No information regarding the prognostic value are present	[66]
	I. ELISA on sera obtained from 36 gastric cancer patients and 9 healthy donors	I. LGALS3BP concentrations are significantly higher in gastric cancer patients compared to healthy blood donors (p = 0.0012). Moreover, higher serum levels positively correlate with distant metastasis occurrence and with tumor stage (p = 0.05 and p = 0.04, respectively)	[66]
Lymphoma (NHL: 2.8%, 2.6%; HL: (0.4%, 0.2%)	I. Sandwich-type ELISA on serum samples collected from 137 patients with lymphoma diagnosed and treated [116 with non-Hodgkin lymphoma (NHL) and 21 with Hodgkin lymphoma (HL)], and 50 control serum samples from blood donors	I. Serum LGALS3BP levels in patients with NHL are significantly higher compared to healthy blood donors (p < 0.001), but not compared to patients with HL (p = 0.2). Higher serum LGALS3BP concentrations significantly correlate with bone marrow involvement in patients with NHL (p = 0.04); and it represents a good marker of treatment response prediction (p = 0 .011)	[67]
Colon carcinoma (6.0%, 5.8%)	I. IHC analysis of 196 paraffin-embedded, archival primary colorectal cancer tissues	I. High LGALS3BP expression correlates with a higher DFS rate (p = 0.011) and with a longer OS (p < 0.002)	[80]
	I. ELISA on serum samples obtained from 20 AD (adenoma) and 20 ADK (adenocarcinoma) patients, as well as from 15 healthy donors II. ELISA on serum samples from 198 CRC patients who received colon cancer resection, of which 174 without clinically detectable metastasis and 24 with liver metastasis	I.Significantly higher levels of LGALS3BP protein are present in AD and ADK patients compared to healthy donors (AD: p = 0.009; ADK: p = 0.0001). No information regarding the prognostic value are present II. High LGALS3BP expression correlates with tumor stage and size (p = 0.014 and p < 0.001, respectively)	[68] [73]
Mesothelioma (0.2%, 0.3%)	I. ELISA on serum samples and PEs (pleural effusions) from 57 cases, of which 28 diagnosed as MM, 14 as LC (lung cancer) and 15 as BPD (benign pleural disease)	I. LGALS3BP level in PEs is significantly higher in MM patients compared to LC (p = 0.011) or BPD (p = 0.021) patients, while no significant differences is detected (p > 0.05) in mean serum LGALS3BP levels between the three groups. LGALS3BP serum levels above the cut off level (7.3 mg/ml) correlates with increased survival probability (p < 0.05)	[81]
Ewing’s sarcoma	I. IHC analysis of TMA with formalin-fixed paraffin embedded tumor specimens from 274 Ewing’s sarcoma patients	I. LGALS3BP expression is associated with better EFS (Event-Free Survival) and OS (p = 0.04 and p = 0.03, respectively)	[82]
	I. ELISA on serum samples from 75 Ewing’s sarcoma patients	I. No association between LGALS3BP levels and prognosis is observed	[82]

### LGALS3BP relevance in cancer-derived extracellular vesicles

Over the past 10 years, attention of scientists has focused on a particular strategy used from cells to exert bidirectional communication with their microenvironment, involving extracellular vesicle (EV) trafficking. EVs are spherical particles delimited by a phospholipid bilayer produced by several eukaryotic cell types according to different physiological and even pathological conditions (i.e. stem cell differentiation, tissue regeneration and angiogenesis, cell activation, changes in pH, hypoxia, irradiation, exposure to complement protein and cellular stress), and secreted into the extracellular microenvironment [90, 91]. Although EVs were initially considered to include waste of the cells, many studies unveiled cell-derived biomolecules like DNA, many RNA types, proteins and lipids, as their cargo. One of the major pathological condition in which EVs played an important role is cancer, where they are involved in a wide range of ‘hallmarks of cancer’ [92], and are crucial in modulating tumor microenvironment in pro-tumoral fashion, becoming useful for the development of cancer diagnostics and therapeutics [90, 91].

First evidence indicating LGALS3BP as one of most abundant EV-enriched protein come from the work by Escrevente et al. in ovarian cancer cell line SKOV-3 [42]. In this study authors proposed that LGALS3BP could play an important role in exosome/target cell interaction via components of the extracellular matrix [42]. Successively, during a large proteomic study analyzing sixty cell lines from the National Cancer Institute (NCI-60), 6071 proteins were identified, including LGALS3BP, probably involved in vesicular packaging and trafficking [93].

EVs-enriched LGASL3BP was also found in neuroblastoma. Indeed, Nakata et al. showed that LGALS3BP was associated with exosome-rich preparations from NB cells obtained with differential ultracentrifugation (DUC), OptiPrep density gradient centrifugation (ODGC) or size exclusion chromatography (SEC) [94]. Intriguing, it has been proposed a functional role for LGALS3BP expressed at the surface of neuroblastoma-derived exosomes, that is the ability to induce the production of IL-6 in a RAS/MEK/ERK-dependent manner. According with these findings, LGALS3BP has been included as functional component of Extracellular vesicles by the International Society for Extracellular Vesicles [95].

Further data suggesting a LGALS3BP role in cancer-derived EVs were obtained in endometrial cancer (EC). One study used a proteomic approach to characterize an epithelial-like population of tumor small EVs originated from the predominant epithelial component of tumor lesions, which might mediate the interaction between Circulating Tumor Cells (CTCs) and microenvironment during the process of endometrial tumor dissemination [96]. As part of this study, LGALS3BP was found to be enriched in circulating EVs isolated from endometrial cancer patients with a high risk of recurrence [96]. A second report confirmed importance of LGALS3BP in EC-derived exosomes, trough proteomic analysis and ELISA revealing that plasma exosomal LGALS3BP increased during EC progression, and it promoted cancer cell growth and angiogenesis via PI3K/AKT/VEGFA signaling [97]. Moreover, high LGALS3BP expression was found to correlate with VEGFA expression and vessel density, indicating a contribution of LGALS3BP in EC development and progression [97].

Castillo et al. [98] identified LGALS3BP as a PDAC-specific biomarker, analyzing a panel of PDAC-specific exosomal surface proteins (“surfaceome”) in 13 human PDACs and 2 non-neoplastic cell lines by liquid chromatography/mass spectrometry.

In a recent study, the colon cancer cell line SW480-derived exosome surfaceome was analyzed using gentle proteolytic digestion (proteinase K), which cleaved surface-exposed proteins. Authors found that LGALS3BP is one of the protein localized on the outer surface of exosomes, possibly involved in interaction with CD9 and CD82 [98].

Finally, a work from Zhang et al. [99], identified LGALS3BP associated with a nonmembranous EV subpopulation called “exomeres”, co-isolated with small EVs at high-speed ultracentrifugation. Exomeres were proposed to mediate specific interactions through adhesion molecules identified in EVs as collagens, fibronectin, nidogen, galectin-3 and fibronectin β1.

### LGALS3BP: a potential therapeutic target in cancer

As in depth described up to here, an impressive body of results from the past two decades have been accumulated demonstrating LGALS3BP as an indisputable player in tumor progression and development of metastasis. Moreover, elevated expression levels of LGALS3BP in serum and tumor tissue of cancer patients have been found and positively correlated with a poor survival or a more advanced and/or metastatic disease in the large majority of solid human cancers.

The numerous pro-tumoral mechanisms in which LGALS3BP is involved are related to its multidomain nature and the association with different ligands, and include adhesion, migration, angiogenesis, motility and immune response (Fig. 2). More recently, it was also clear that extracellular vesicles—associated LGALS3BP is a key regulator in cell–cell and cell-extracellular matrix cross-talk in the cancer context [42, 94, 97]. Therefore, as summarized in this review behind the notion that LGALS3BP may serve as an attractive therapeutic target in cancer, there is a strong rationale. A first report from our group demonstrated that the murine monoclonal antibody, named SP2, recognizing a conformational epitope of the lectin-binding domain of LGALS3BP [6, 33], is able to reduce LGALS3BP-induced tube formation in Matrigel by endothelial cells [18]. Successively, we investigated LGALS3BP as therapeutic target through the use of SP2 antibody which showed the ability to antagonize LGALS3BP-induced endothelial cells tubulogenesis in vitro [100]. Moreover, in the same work, it was reported that the therapeutic murine antibody was able to inhibit angiogenesis to the same extent of bevacizumab using a Matrigel plug model containing MDA-MB-231 human breast cancer cells. Although the antibody did not affect tumor cell growth in vitro, it led to a significant growth delay in several xenograft models, including breast, ovarian carcinoma and particularly melanoma, associated with a reduced number of blood vessels as evidenced by CD31/CD105 staining, indicating that the antibody targets tumor vasculature in vivo [100].

More recently, our group attempted to evaluate the role of LGALS3BP as a drug target using a novel immunotherapy approach, based on Antibody–Drug Conjugates (ADC)s, which have emerged as a promising strategy for the development of efficient therapy in several malignancies. Indeed, while initial trials of ADC focused on tumor associated antigens or tumor growth factor receptors expressed on the surface of tumor cells, more recently stromal components of the tumour microenvironment have been explored as potentially actionable targets. In this context, we have recently focused our attention to the development of a new type of non-internalizing ADC [101, 102]. We hypothesize that this ADC might work by recognizing the antigen expressed both on the EVs surface and in the extracellular matrix, releasing the drug in the reducing space of tumor micro-environment, resulting in a potent therapeutic effect as described below.

A humanized version of the murine SP-2 antibody, named 1959 was generated and successively engineered through cysteine to serine substitution into the hinge region (hereafter named 1959-sss) allowing a site-specific, linker-less thiol-drug coupling at the residual C-terminal cysteines of the light chain. This procedure ensured product homogeneity with a defined DAR of 2. Three 1959-sss/based ADC products were obtained using as payloads the maytansinoid thiol-derivatives DM1-SH, DM3-SH and DM4-SH and tested on LGALS3BP^+^ melanoma xenograft model. The therapeutic study showed that 1959-sss/DM3 and 1959-sss/DM4 possess a potent antitumor activity with induction of long-lasting complete remission [101].

More recently, in light of new findings on the abundance of LGALS3BP in extracellular vesicles in several cancers, we investigated the therapeutic potential of 1959-sss/DM3 in multiple pre-clinical models of human neuroblastoma expressing vesicular LGALS3BP [102]. This work proved that ADC-therapy targeting LGALS3BP in neuroblastoma induced significant shrinkage of established subcutaneous and orthotopic neuroblastomas, as well as inhibition of metastatic dissemination and complete eradication of subcutaneous high-risk neuroblastoma patient-derived xenografts (PDX)s harboring MYCN amplification.

## Conclusions and future directions

LGALS3BP is a ubiquitous hyper-glycosylated protein which is found intracellularly and body fluids. The presence in its structure of different domains combined with its ability to interact with a variety of signaling molecules, make this protein a primary player in a variety of cellular mechanisms, ranging from innate immune responses and inflammation to tumor progression and metastatic spreading. To make the physiological role of this protein even more complex and interesting, is the recent evidence that LGALS3BP is one of the most represented proteins in the vesicular compartments of cancer cells, the so-called extracellular vesicles (EV)s.

A large body of data have been collected so far, which has indubitably revealed the role of LGALS3BP as a prognostic marker in a variety of cancer disease. Moreover, given its ability to participate in several pro-tumoral mechanisms, the notion that it may serve as therapeutic target has strongly emerged. In line with this, using an innovative ADC-based therapy, our group has recently opened the way to anti-LGALS3BP therapy highlighting the potential of this molecule as target for cancer therapy. The high antitumor activity observed it is thought to occur because of the vast accumulation of LGALS3BP in the tumor allowing accumulation of the therapeutic ADC at the site of the disease. On the other hand, the potential risk of using LGALS3BP as therapeutic target is linked to its nature of secreted protein. Indeed, relative high amount of circulating LGALS3BP can be detected in serum and this may affect the efficacy of a therapeutic ADC due to a potential sink effect. This possibility is currently being investigated.

In conclusion, further studies are needed to fully exploit the potential of LGALS3BP as a diagnostic marker, a driver of malignancy and progression as well as a therapeutic target in cancer.

## Data Availability

Not applicable.
